# RNA-Based Therapeutics in Genetic Neurodevelopmental Disorders: Bridging Molecular Genetics and Precision Medicine

**DOI:** 10.3390/ijms27177725

**Published:** 2026-08-28

**Authors:** Ina-Ofelia Focșa, Catrinel Iliescu, Cristina Pomeran, Magdalena Budișteanu, Carmen Sandu, Alice Denisa Dică, Florentina Ionela Lincă, Cristina Moțoiescu, Diana Bârcă, Ioana Minciu, Dana Craiu, Viorica Elena Rădoi

**Affiliations:** 1 Department of Medical Genetics, Faculty of Medicine, Carol Davila University of Medicine and Pharmacy, 020021 Bucharest, Romania; florentina-ionela.linca@fpse.unibuc.ro (F.I.L.); viorica.radoi@umfcd.ro (V.E.R.); 2 Psychiatry Research Laboratory, Prof. Dr. Alexandru Obregia Clinical Hospital of Psychiatry, 041914 Bucharest, Romania; magda.budisteanu@ivb.ro; 3 Pediatric Neurology Department, Faculty of Medicine, Carol Davila University of Medicine and Pharmacy, 041914 Bucharest, Romania; catrinel.iliescu@umfcd.ro (C.I.); cristina.pomeran@umfcd.ro (C.P.); carmen.sandu@umfcd.ro (C.S.); alice.dica@drd.umfcd.ro (A.D.D.); cristina.motoiescu@umfcd.ro (C.M.); diana.barca@umfcd.ro (D.B.); ioana.minciu@umfcd.ro (I.M.); dana.craiu@umfcd.ro (D.C.); 4 Pediatric Neurology Department, Prof. Dr. Alexandru Obregia Clinical Hospital of Psychiatry, 041914 Bucharest, Romania; 5 Medical Genetics Laboratory, Victor Babes National Institute of Pathology, 050096 Bucharest, Romania; 6 Department of Genetics, Faculty of Medicine, Titu Maiorescu University, 031593 Bucharest, Romania; 7 Department of Special Psychopedagogy, Faculty of Psychology and Educational Sciences, University of Bucharest, 0506578 Bucharest, Romania; 8 “Alessandrescu-Rusescu” National Institute for Maternal and Child Health, 20382 Bucharest, Romania

**Keywords:** RNA therapeutics, neurodevelopmental disorders, antisense oligonucleotides, precision medicine, spinal muscular atrophy, Rett syndrome, Angelman syndrome, RNA editing, pediatric neurogenetics, personalized medicine

## Abstract

Genetic neurodevelopmental disorders (NDDs) encompass a heterogeneous group of conditions characterized by impaired cognitive, behavioral, and neurological development resulting from pathogenic variants affecting brain development and synaptic function. Advances in molecular genetics and next-generation sequencing have significantly expanded the understanding of the genetic architecture underlying disorders such as Rett syndrome (RTT), Fragile X syndrome (FXS), Angelman syndrome (AS), and autism spectrum disorders. Beyond these classical neurodevelopmental disorders, spinal muscular atrophy (SMA) is included as a paradigmatic example of successful RNA-based therapeutic translation. Concurrently, RNA-based therapeutics have emerged as promising precision medicine strategies capable of modulating gene expression at the transcriptional and post-transcriptional levels. These approaches include antisense oligonucleotides (ASOs), small interfering RNAs (siRNAs), messenger RNA (mRNA) therapies, RNA editing technologies, and splice-modulating agents. Recent clinical successes, particularly in spinal muscular atrophy, have demonstrated the transformative potential of RNA therapeutics in neurological disease. However, substantial challenges remain, including BBB penetration, long-term safety, immune activation, and genotype-specific variability in therapeutic response. This review summarizes current advances in RNA-based therapeutics for genetic NDDs, highlighting molecular mechanisms, disease-specific therapeutic strategies, translational progress, delivery challenges, and future directions. Overall, continued progress will depend on the integration of disease biology, rational RNA therapeutic design, and effective CNS-targeted delivery, supporting the broader implementation of precision RNA medicine for genetic neurodevelopmental disorders.

## 1. Introduction

Neurodevelopmental disorders (NDDs) comprise a heterogeneous group of conditions characterized by impairments in cognitive, behavioral, motor, and social functioning resulting from abnormal brain development. These disorders include both common neurodevelopmental conditions, such as intellectual disability (ID), autism spectrum disorder (ASD), and developmental and epileptic encephalopathies, and gene- or chromosomal locus-specific syndromes, including Rett syndrome (RTT), Fragile X syndrome (FXS), and Angelman syndrome (AS) [1]. Beyond these classical neurodevelopmental disorders, advances in RNA therapeutics have also been driven by neurological diseases with shared molecular and translational challenges, most notably spinal muscular atrophy (SMA). Collectively, NDDs affect approximately 1–4% of the global population and represent a major cause of lifelong disability, with profound consequences for affected individuals, their families, and healthcare systems [2].

Recent advances in genomic technologies, particularly next-generation sequencing (NGS), whole-exome sequencing (WES), and whole-genome sequencing (WGS), have revolutionized the understanding of the molecular basis of NDDs. Thousands of genes have been implicated in neurodevelopmental processes, including neuronal proliferation, migration, synaptogenesis, chromatin remodeling, transcriptional regulation, and intracellular signaling. The increasing identification of pathogenic variants has transformed many previously idiopathic neurodevelopmental conditions into genetically defined disorders, shifting clinical practice toward genotype-driven diagnosis and creating new opportunities for mechanism-based therapeutic intervention [3,4,5].

Despite remarkable progress in molecular diagnosis, therapeutic options for most genetic NDDs remain limited and are largely focused on symptomatic management. Conventional pharmacological approaches remain essential for controlling seizures, behavioral manifestations, sleep disturbances, and other clinical complications, but they rarely address the molecular defects responsible for disease pathogenesis or substantially modify the underlying disease process. Increasingly, identification of causal variant informs not only diagnosis and prognosis but also therapeutic target selection and the choice of an appropriate molecular intervention [6,7].

RNA-based therapeutics have emerged as a promising class of precision medicine approaches capable of modulating gene expression without permanent modification of the genome. These approaches encompass a diverse spectrum of transcript-directed strategies, including splice modulation, transcript silencing, messenger RNA (mRNA) replacement, RNA activation, and RNA editing, each suited to distinct pathogenic mechanisms at the RNA level. Some RNA-directed approaches may employ viral vectors as delivery vehicles, yet their therapeutic effect remains mediated through modulation of RNA rather than permanent modification of genomic DNA. The functional consequences of pathogenic variants provide the mechanistic link between molecular diagnosis and therapeutic selection: splice-altering variants may be addressed through splice modulation, toxic gain-of-function transcripts through transcript silencing, haploinsufficiency through transcript enhancement or mRNA replacement, and selected nucleotide substitutions through RNA editing. Therapeutic platform selection is therefore increasingly guided by the underlying molecular defect rather than by the clinical phenotype alone. The clinical success of nusinersen for SMA provided the first compelling evidence that precise manipulation of RNA biology can substantially alter the natural history of a severe neurogenetic disorder and established a foundation for the development of similar therapies across a broad range of genetic diseases [8].

The central nervous system (CNS) presents unique opportunities and challenges for RNA therapeutics. Many NDDs arise from highly specific molecular defects that are potentially amenable to targeted intervention, yet successful clinical translation requires adequate biodistribution throughout relevant regions of the brain and spinal cord, uptake by the appropriate neuronal or glial populations, efficient intracellular trafficking and endosomal escape, and sustained therapeutic exposure. Current routes of administration may not achieve uniform tissue penetration or therapeutically effective concentrations across all relevant CNS regions. An additional challenge lies in the temporal dimension of neurodevelopment. For many genetic disorders, pathogenic mechanisms begin during prenatal or early postnatal brain development, whereas clinical diagnosis frequently occurs only after symptoms have become established. The therapeutic window may therefore differ substantially across disorders, and molecular correction after symptom onset may not fully reverse neurodevelopmental abnormalities that have already emerged. Overcoming these barriers while maintaining cellular specificity and long-term safety remains central to the clinical development of RNA medicine for NDDs [9].

A key knowledge gap lies in translating the rapidly expanding spectrum of RNA therapeutic technologies into a disease-specific framework that links the underlying molecular defect to therapeutic modality selection, CNS delivery requirements, and the level of translational readiness. Addressing this gap is particularly relevant for genetically heterogeneous NDDs, in which the feasibility and maturity of RNA-based intervention vary substantially according to disease mechanism and therapeutic target. This review provides a comprehensive overview of the emerging role of RNA therapeutics in genetic NDDs through the perspective of precision medicine. It integrates molecular genetics, therapeutic platform selection, CNS delivery, and translational readiness within a unified precision medicine framework. The primary focus is on monogenic NDDs and genetically defined disease mechanisms that are amenable to transcript-directed intervention. SMA is included as a paradigmatic neurological disease whose successful clinical translation established the proof of concept for CNS-directed RNA therapeutics. We first discuss the molecular basis of RNA dysregulation and major RNA therapeutic platforms before reviewing their application across representative NDDs. We then examine current strategies for CNS delivery, followed by the principal translational challenges and future directions that will determine the successful clinical implementation of RNA-based therapies.

## 2. Material and Methods

A comprehensive literature search was conducted in PubMed, Scopus, and Web of Science to identify relevant publications on RNA-based therapeutics for genetic neurodevelopmental disorders. Searches included combinations of keywords related to RNA therapeutics (“antisense oligonucleotides”, “ASOs”, “siRNA”, “mRNA”, “RNA editing”, “RNA therapeutics”), neurodevelopmental disorders (“neurodevelopmental disorders”, “autism spectrum disorder”, “intellectual disability”, “developmental and epileptic encephalopathies”), and representative disease-associated genes discussed throughout the review (including *MECP2*, *UBE3A*, *FMR1*, *SYNGAP1*, *SCN2A*, *KCNT1*, and *SMN1*). Original articles, preclinical studies, clinical trials, systematic reviews, and high-quality review articles published in English were considered, with no lower publication-date restriction applied. The final literature search was conducted on 30 June 2026, with additional targeted searches performed during manuscript revision to identify newly published studies directly relevant to the therapeutic strategies discussed. Priority was given to recent studies, while landmark publications were included to provide historical context and support discussion of key therapeutic developments. Disorders were selected to represent genetically defined conditions with well-characterized molecular mechanisms and established, clinical, preclinical or emerging evidence supporting the potential for RNA-directed therapeutic intervention. Therapeutic platforms were included when their mechanism of RNA modulation was directly relevant to the underlying disease biology, including splice modulation, transcript suppression or enhancement, mRNA replacement, and RNA editing. As this is a narrative review, no formal systematic review protocol or meta-analysis was undertaken.

## 3. Molecular Genetics of Neurodevelopmental Disorders

### 3.1. Genetic Architecture of NDDs

NDDs exhibit remarkable genetic heterogeneity, encompassing monogenic, chromosomal, and complex polygenic etiologies.

Advances in genomic technologies and large-scale sequencing studies have substantially expanded our understanding of NDD etiology, revealing important contributions from both inherited and de novo genetic variants [5]. Large-scale sequencing studies have demonstrated that both inherited and de novo variants contribute substantially to disease risk, with de novo mutations accounting for a significant proportion of severe developmental disorders and intellectual disabilities [10,11].

The genes implicated in NDDs participate in diverse biological pathways essential for brain development and function [12]. These include regulators of chromatin remodeling (*MECP2*, *CHD8*, *ARID1B*) [13,14], synaptic structure and signaling (*SHANK3*, *SYNGAP1*, *NRXN1*) [15,16], ion channel function (*SCN1A*, *SCN2A*, *KCNQ2*) [17,18], protein translation (*FMR1*) [19], ubiquitin-mediated protein degradation (*UBE3A*) [20], and RNA processing mechanisms, including alternative splicing, RNA transport, stability, and translation [21]. Disruption of these pathways can impair neuronal differentiation, synaptic plasticity, network formation, and circuit maturation, ultimately leading to neurodevelopmental phenotypes [12].

The increasing identification of monogenic causes of NDDs has not only improved diagnostic precision but has also created opportunities for targeted therapeutic interventions. In particular, disorders resulting from haploinsufficiency, loss-of-function variants, aberrant splicing, or epigenetic silencing may be suitable candidates for RNA-based therapeutic approaches aimed at restoring physiological gene expression [10,22,23].

### 3.2. RNA Dysregulation as a Therapeutic Target in Neurodevelopmental Disorders

The human brain exhibits one of the most complex transcriptomic landscapes among all organs, characterized by extensive alternative splicing, dynamic RNA editing, tightly regulated mRNA localization, and activity-dependent translation [21,24]. These RNA-mediated regulatory mechanisms are essential for neuronal differentiation, synapse formation, circuit maturation, and long-term synaptic plasticity. Consequently, disturbances in RNA metabolism have emerged as central pathogenic mechanisms in numerous neurodevelopmental disorders (NDDs) [19,25,26].

Alternative splicing represents a particularly important source of transcriptomic diversity within the nervous system. It is estimated that more than 90% of multi-exonic human genes undergo alternative splicing, with the highest complexity observed in neuronal tissues [21,24,27].

Precise spatiotemporal regulation of exon inclusion and exclusion is required for normal neurodevelopment, and disruptions in these processes may result in altered protein isoforms with impaired function [24,28]. Aberrant splicing has been implicated in several neurodevelopmental and neurogenetic disorders; a paradigmatic example is SMA, where exclusion of exon 7 from the survival motor neuron 2 (*SMN2*) transcript leads to reduced levels of functional survival motor neuron (SMN) protein [29,30].

Beyond splicing abnormalities, defects in RNA transport and local translation contribute significantly to neurodevelopmental pathology. Neurons rely on the targeted transport of mRNA molecules to dendrites and axons, where local protein synthesis enables rapid responses to synaptic activity [25,31]. FXS exemplifies this mechanism, as loss of fragile X messenger ribonucleoprotein (FMRP) disrupts translational regulation at synapses, resulting in excessive protein synthesis and impaired synaptic plasticity [19,32]. Similar disturbances have been identified in autism spectrum disorders (ASDs) associated with mutations affecting translational control pathways, including components of the mTOR signaling cascade [33,34].

RNA stability and degradation pathways also play crucial roles in neuronal homeostasis. Dysregulation of nonsense-mediated mRNA decay (NMD), microRNA-mediated repression, and RNA-binding proteins can alter neuronal gene expression profiles and contribute to disease pathogenesis [35,36]. Mutations in genes encoding RNA-binding proteins have been associated with developmental delay, intellectual disability, epilepsy, and autism-related phenotypes, further highlighting the importance of post-transcriptional regulation during brain development [37,38].

Epigenetic mechanisms influencing RNA expression provide an additional layer of complexity [39]. Angelman syndrome, for example, results from loss of maternal UBE3A expression in neurons, while the paternal allele remains epigenetically silenced by a long antisense transcript (UBE3A-ATS) [20,40]. This unique regulatory architecture has created an attractive therapeutic opportunity for RNA-targeted approaches capable of unsilencing the paternal allele and restoring endogenous protein production [40,41].

The growing recognition that many NDDs arise from defects affecting RNA processing, stability, or regulation has transformed RNA molecules from passive intermediates into active therapeutic targets [42,43]. Unlike conventional pharmacological strategies that often attempt to compensate for downstream consequences of genetic defects, RNA-based therapies can intervene closer to the primary molecular cause of disease [20,44]. This capacity for precise modulation of gene expression forms the foundation of modern RNA medicine and explains its increasing relevance in the treatment of NDDs [45,46,47].

## 4. RNA-Based Therapeutic Platforms

### 4.1. Antisense Oligonucleotides

Antisense oligonucleotides (ASOs) are short, synthetic single-stranded nucleic acids, typically 15–30 nucleotides in length, designed to bind complementary RNA sequences through Watson–Crick base pairing [44,48]. By selectively interacting with target transcripts, ASOs can modulate gene expression at multiple stages of RNA processing, making them one of the most versatile platforms within the field of RNA therapeutics. Depending on their design and target site, ASOs may induce RNase H-mediated transcript degradation, alter pre-mRNA splicing, inhibit translation, stabilize RNA molecules, or regulate the expression of endogenous genes [48,49].

The therapeutic properties of ASOs have been greatly enhanced by advances in oligonucleotide chemistry. First-generation phosphorothioate backbone modifications improve resistance to nuclease degradation and prolong tissue retention, while second- and third-generation sugar modifications, including 2′-O-methoxyethyl (2′-MOE), 2′-O-methyl (2′-OMe), and locked nucleic acids (LNAs), increase target affinity and improve pharmacokinetic profiles. These modifications have enabled efficient distribution within the central nervous system (CNS) following intrathecal administration and have contributed to the clinical success of ASO-based therapies for neurological disorders [44,50,51].

Several distinct therapeutic strategies have emerged from ASO technology. One major application involves splice modulation, whereby ASOs bind regulatory elements within pre-mRNA to promote or inhibit exon inclusion [52]. This approach can restore normal transcript processing in diseases caused by aberrant splicing or can be used to modify protein structure through controlled exon skipping [44,53]. The most prominent example is nusinersen, which promotes inclusion of exon 7 in the SMN2 transcript and increases production of functional SMN protein. Clinical studies demonstrated substantial improvements in motor function, survival, and developmental outcomes in patients with SMA, establishing a landmark proof-of-concept for RNA-based therapies in pediatric neurology [52,54,55].

ASOs can also be designed to induce degradation of target transcripts through recruitment of RNase H, an endogenous enzyme that cleaves RNA strands within RNA–DNA heteroduplexes. This strategy is particularly attractive for disorders caused by toxic gain-of-function mutations, dominant-negative alleles, or pathological gene overexpression, as selective reduction in disease-causing transcripts may alleviate downstream cellular dysfunction [44,48,56]. In parallel, advances in allele-specific ASO design have enabled selective targeting of mutant transcripts while preserving expression of the corresponding wild-type allele, thereby expanding therapeutic opportunities for dominantly inherited neurological disorders, with increasing applications in NDDs [44,57].

Another important application involves restoration of endogenous gene expression. Rather than suppressing target transcripts, some ASOs are designed to interfere with naturally occurring antisense RNAs or other cis-regulatory RNA elements that repress gene activity. By removing these inhibitory mechanisms, ASOs can reactivate functional copies of genes that remain intact but transcriptionally silent. This strategy has generated considerable interest for disorders characterized by haploinsufficiency or epigenetic dysregulation, where even partial restoration of endogenous gene expression may be sufficient to produce clinically meaningful benefit [58,59,60].

The versatility of ASO technology has stimulated its investigation across a broad range of NDDs. In Angelman syndrome, ASOs targeting the *UBE3A* antisense transcript aim to unsilence the paternal *UBE3A* allele and restore neuronal protein expression. Similar gene-reactivation approaches are being explored preclinically for other disorders involving dosage-sensitive genes [40,41,61,62]. In RTT, preclinical strategies targeting regulatory pathways that influence *MECP2* expression seek to achieve controlled normalization of protein levels, while ASO-based approaches for *SCN2A*, *SCN8A*, and *KCNT1*-related disorders aim to reduce expression of pathogenic gain-of-function alleles associated with developmental and epileptic encephalopathies [63,64,65,66]. Likewise, haploinsufficiency disorders may benefit from ASO-mediated enhancement of endogenous gene expression, although applications to SYNGAP1-related intellectual disability and related dosage-sensitive NDDs remain at an early preclinical stage [62].

Despite their considerable promise, several challenges continue to limit the broader implementation of ASO therapies in NDDs. Most ASOs require repeated intrathecal administration because they do not readily cross the BBB, and achieving uniform distribution throughout the CNS remains difficult. Therapeutic efficacy may also depend on intervention during critical developmental windows, raising important questions regarding treatment timing and long-term safety. Furthermore, off-target hybridization, inflammatory responses, and variable cellular uptake remain areas of active investigation. Ongoing efforts to improve oligonucleotide chemistry, tissue targeting, and delivery systems are expected to further enhance the safety and efficacy of ASO-based therapies [9,56,59].

### 4.2. Small Interfering RNAs

Small interfering RNAs (siRNAs) are synthetic or endogenous double-stranded RNA molecules, typically 20–25 nucleotides in length, that induce sequence-specific post-transcriptional gene silencing through the endogenous RNA interference (RNAi) pathway. Following cellular uptake, siRNAs are incorporated into the RNA-induced silencing complex (RISC), where the passenger strand is removed and the guide strand directs the complex to complementary mRNA targets. Binding of the siRNA-loaded RISC promotes cleavage and degradation of the target transcript, resulting in reduced protein expression [67,68,69,70,71].

Because siRNAs act through selective transcript degradation, they are particularly well suited for disorders driven by toxic gain-of-function mutations, dominant-negative variants, or pathological gene overexpression. Their high sequence specificity enables targeted suppression of disease-associated transcripts while minimizing effects on unrelated genes. In addition, advances in RNA chemistry, including nucleotide modifications and conjugation strategies, have improved siRNA stability, pharmacokinetic properties, and resistance to nuclease degradation, supporting the successful clinical development of several siRNA-based therapies for non-neurological diseases [72,73,74].

The ability to selectively reduce pathogenic gene expression makes siRNAs an attractive therapeutic strategy for genetic NDDs, particularly those caused by dominant disease mechanisms. Unlike gene replacement approaches, which are primarily applicable to loss-of-function disorders, siRNAs can directly suppress the expression of pathogenic alleles. Allele-specific silencing strategies have therefore attracted considerable interest, as they enable selective inhibition of mutant transcripts while preserving expression of the wild-type allele [42,68]. This approach may be especially relevant for developmental and epileptic encephalopathies associated with gain-of-function variants in genes encoding ion channels and neuronal signaling proteins. Genes such as *SCN2A*, *SCN8A*, and *KCNT1* have been proposed as potential targets for allele-specific RNA interference approaches aimed at reducing neuronal hyperexcitability and restoring network function [6,66,75].

Beyond direct suppression of mutant genes, siRNAs may also be used to modulate molecular pathways that contribute to disease pathogenesis. Experimental studies have shown that RNA interference can regulate components of signaling networks involved in synaptic function, excitatory-inhibitory balance, neuroinflammation, and neuronal survival. Such approaches could complement gene-specific therapies by targeting downstream mechanisms shared across multiple NDDs [26,76].

Clinical implementation of siRNA therapeutics in neurological disorders is currently constrained by several important challenges. Efficient delivery to the central nervous system (CNS) remains a major obstacle because of the BBB, limited cellular uptake of naked RNA molecules, and the need for broad distribution across relevant neuronal populations. Notably, most approved siRNA therapies rely on efficient delivery to the liver, whereas selective targeting of neurons and other CNS cell types remains considerably more challenging. Additional concerns include off-target silencing, immune activation, heterogeneous tissue distribution, and the potential requirement for repeated administration to maintain long-term therapeutic effects. To address these limitations, extensive efforts are focused on developing advanced delivery platforms, including lipid nanoparticles, polymer-based carriers, viral vector-mediated systems, and extracellular vesicles [9,68,76].

### 4.3. Messenger RNA Therapeutics

Messenger RNA-based therapeutics represent a rapidly emerging class of nucleic acid medicines designed to restore protein expression through the delivery of synthetic coding transcripts. Unlike viral gene replacement approaches, mRNA therapies do not require nuclear entry or genomic integration, thereby avoiding the risk of insertional mutagenesis and enabling controlled, transient protein production. These characteristics make mRNA an attractive therapeutic strategy for disorders caused by loss-of-function mutations, haploinsufficiency, or protein deficiency [77,78].

The therapeutic principle of mRNA-based interventions is based on the delivery of synthetic mRNA molecules encoding a functional protein of interest. Following uptake into target cells, the mRNA is translated by the host cellular machinery, resulting in production of the encoded protein. Recent advances in RNA engineering, including codon optimization, incorporation of modified nucleosides, and refinement of untranslated regions (UTRs), have substantially improved mRNA stability, translational efficiency, and tolerability. In parallel, the development of lipid nanoparticle (LNP) delivery systems has enhanced intracellular delivery and protected mRNA molecules from enzymatic degradation, facilitating the clinical translation of this technology [79,80,81].

The success of mRNA vaccines during the COVID-19 pandemic accelerated interest in broader therapeutic applications, including rare genetic diseases and neurological disorders. In the context of NDDs, mRNA therapeutics offer a particularly attractive approach for conditions in which restoration of protein expression may be sufficient to improve cellular function. By supplying a functional transcript, mRNA therapies have the potential to compensate for deficient gene activity without permanently altering the genome, providing a reversible alternative to DNA-based gene replacement strategies [81,82].

Several NDDs could theoretically benefit from mRNA-mediated protein replacement. RTT, caused by pathogenic variants in *MECP2*, represents a potential target, although the narrow therapeutic window associated with *MECP2* dosage necessitates precise regulation of protein expression [83,84]. Similarly, disorders arising from haploinsufficiency of genes such as *SYNGAP1*, *SHANK3*, and selected loss-of-function *SCN2A* variants have been proposed as potential candidates for mRNA-mediated protein replacement. However, direct preclinical evidence supporting this therapeutic approach has yet to be established [85]. The modular nature of mRNA design further supports the development of personalized therapeutic approaches by enabling rapid adaptation to diverse disease-causing variants.

The application of mRNA therapeutics to disorders affecting the central nervous system (CNS) remains limited by several important challenges. The large size and inherent instability of mRNA molecules complicate systemic delivery, while efficient transport across the BBB remains a major obstacle. In addition, the transient nature of mRNA expression may necessitate repeated administration to maintain therapeutic benefit. Other concerns include immune activation, variability in protein expression levels, and the need for cell-specific targeting to minimize unintended biological effects [86,87].

To address these limitations, considerable efforts are focused on the development of next-generation delivery platforms capable of improving CNS biodistribution and neuronal uptake. Novel BBB-crossing lipid nanoparticles, biodegradable polymer-based systems, extracellular vesicle-mediated delivery, and receptor-targeted transport strategies are currently being investigated to enhance brain-specific delivery of therapeutic mRNAs. Advances in these technologies are expected to play a critical role in determining the clinical feasibility of mRNA-based interventions for NDDs [88,89].

### 4.4. RNA Editing Technologies

RNA editing technologies have emerged as a promising therapeutic approach for the correction of disease-causing variants at the transcript level. Unlike genome-editing strategies, which permanently modify DNA sequences, RNA editing enables transient and potentially reversible modification of RNA transcripts without altering the underlying genome. This feature is particularly attractive for NDDs, where precise control of gene dosage and long-term safety are critical considerations [90,91].

The most prevalent form of endogenous RNA editing in mammalian cells is adenosine-to-inosine (A-to-I) conversion mediated by adenosine deaminases acting on RNA (ADARs). Because inosine is interpreted as guanosine during translation and RNA processing, ADAR-mediated editing can effectively recode specific nucleotides within messenger RNA transcripts. This naturally occurring mechanism has provided the foundation for the development of programmable RNA editing platforms capable of correcting pathogenic variants at the RNA level [92,93].

Several complementary approaches have been developed to achieve targeted RNA editing. One strategy employs engineered guide RNAs that recruit endogenous ADAR enzymes to specific transcripts, enabling site-directed nucleotide conversion without the need for exogenous protein expression. Alternative platforms utilize fusion proteins that combine RNA-targeting domains with catalytically active deaminases to increase editing efficiency and broaden the range of editable targets. In experimental systems, these technologies have been used to correct nonsense mutations, restore protein function, and modulate gene expression in models of inherited disease [94,95,96].

RNA editing is particularly attractive for NDDs because many monogenic conditions are caused by single-nucleotide variants that could theoretically be corrected at the transcript level. Disorders associated with pathogenic variants in genes such as *MECP2*, *SCN1A*, *SCN2A*, and *UBE3A* have emerged as promising candidates for RNA editing–based therapeutic strategies. By correcting pathogenic transcripts while preserving genomic integrity, RNA editing may provide an alternative to permanent DNA modification in situations where irreversible genome editing raises safety concerns [91,97,98].

RNA-targeting Cas13 systems have further expanded the repertoire of programable RNA technologies. Members of the CRISPR-Cas13 family recognize RNA rather than DNA and can be programmed to selectively bind, degrade, or modify target transcripts. In a mouse model of Angelman syndrome, hfCas13x.1-mediated reduction in the UBE3A antisense transcript restored paternal *UBE3A* expression and improved motor function [99].

Beyond transcript knockdown, engineered Cas13-based platforms have been adapted for RNA editing, transcript stabilization, and regulation of gene expression, further broadening the range of programmable RNA-directed therapeutic strategies [94,97,100].

Several biological and technical barriers must still be overcome before RNA editing can be translated into clinical practice. Efficient and cell type-specific delivery to neuronal populations, maintenance of editing specificity, durability of therapeutic effects, and minimization of off-target modifications remain major challenges for clinical translation. Furthermore, the complexity of neuronal transcriptomes and the genetic heterogeneity of NDDs necessitate highly precise targeting and rigorous evaluation of long-term safety [92,100].

### 4.5. Emerging RNA-Based Approaches

Beyond established platforms such as ASOs, siRNAs, mRNA therapeutics, and RNA editing technologies, a growing number of innovative RNA-based strategies are expanding the therapeutic landscape for NDDs. These approaches exploit diverse aspects of RNA biology, including endogenous gene regulation, transcript stability, and translational control, thereby creating new opportunities to modulate disease-associated molecular pathways [8,80].

MicroRNAs (miRNAs) are endogenous non-coding RNAs that regulate gene expression through post-transcriptional repression of target transcripts. Individual miRNAs can influence complex gene networks involved in neuronal differentiation, synaptic plasticity, and neurodevelopment. Dysregulation of miRNA signaling has been implicated in several NDDs, including ASD, FXS, and RTT. Consequently, therapeutic strategies based on miRNA mimics or anti-miRNA oligonucleotides are being explored to restore physiological regulatory networks and normalize aberrant gene expression profiles [101,102].

Long non-coding RNAs (lncRNAs) have also emerged as important therapeutic targets. These transcripts participate in chromatin remodeling, transcriptional regulation, and epigenetic control of gene expression. A notable example is the *UBE3A* antisense transcript (*UBE3A-ATS*), which mediates silencing of the paternal *UBE3A* allele in AS. Therapeutic modulation of disease-associated lncRNAs may therefore enable reactivation of endogenous genes and restoration of physiologically regulated protein expression without the need for exogenous gene replacement [40,103].

Small activating RNAs (saRNAs) represent another emerging class of therapeutic RNAs that enhance endogenous gene expression through a process known as RNA activation (RNAa). Unlike siRNAs, which suppress gene expression, saRNAs promote transcription by recruiting regulatory complexes to gene promoters, resulting in increased expression of endogenous genes. This strategy may be particularly attractive for haploinsufficiency disorders, where modest increases in physiological gene expression could restore protein levels without introducing exogenous genetic material. Although therapeutic applications in NDDs remain at an early stage, saRNAs represent a promising approach for disorders caused by haploinsufficiency [104,105].

Beyond transcriptional activation, emerging RNA technologies are also exploring strategies to enhance endogenous protein production at the post-transcriptional level. Engineering of untranslated regions (UTRs) and other cis-regulatory RNA elements has emerged as a promising approach to improve mRNA stability, increase translational efficiency, and enhance protein synthesis from dosage-sensitive genes. Such strategies may be particularly attractive for haploinsufficiency disorders, where relatively modest increases in endogenous protein levels could provide meaningful therapeutic benefit. Advances in synthetic biology have further enabled the development of programmable RNA regulators capable of sensing intracellular signals and dynamically controlling gene expression in a context-dependent manner, thereby expanding the therapeutic versatility of RNA medicine [81,106,107].

Additional experimental strategies include circular RNAs (circRNAs), which exhibit enhanced stability compared with linear RNA molecules and may serve as durable platforms for therapeutic protein expression, as well as RNA aptamers capable of selectively binding proteins, nucleic acids, or small molecules to modulate disease-relevant pathways. Although these technologies remain in the early stages of development, they further illustrate the expanding diversity of RNA-based therapeutic modalities [80,108,109].

**Figure 1:**
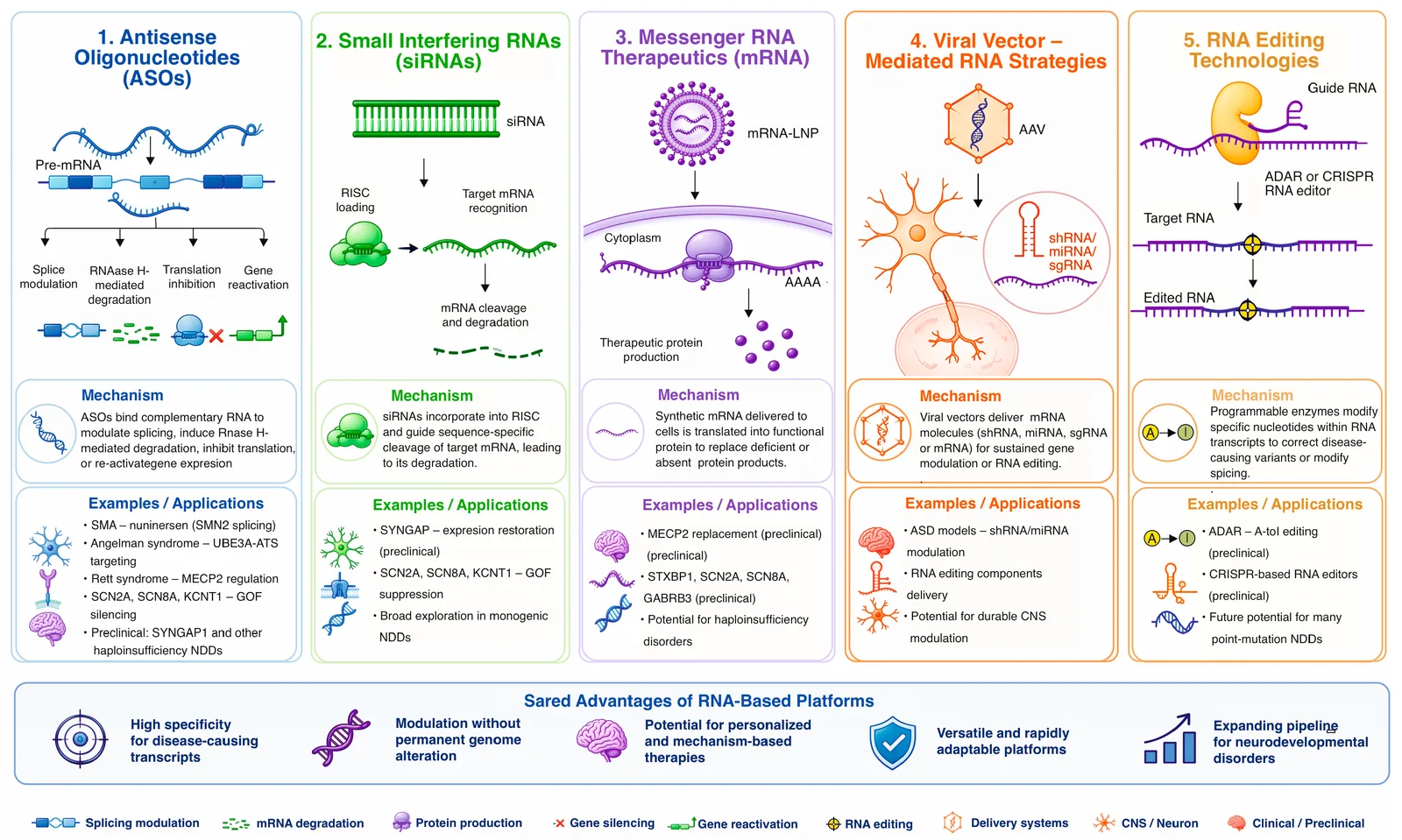
Overview of the major RNA-based therapeutic platforms currently being developed for genetic neurodevelopmental disorders. Antisense oligonucleotides (ASOs), small interfering RNAs (siRNAs), messenger RNA (mRNA) therapeutics, viral vector-mediated RNA strategies, and RNA editing technologies act through distinct molecular mechanisms to modulate gene expression at the transcript level. Each platform offers specific advantages depending on the underlying pathogenic mechanism, including splice correction, transcript degradation, protein replacement, long-term RNA modulation, or precise transcript editing. Together, these complementary technologies constitute the expanding therapeutic toolbox of RNA-based precision medicine.

Collectively, these emerging approaches illustrate the expanding landscape of RNA-based therapeutic strategies for NDDs (Figure 1) [81].

## 5. Disease-Specific Applications of RNA Therapeutics

The therapeutic application of RNA medicines in NDDs is largely determined by the underlying molecular mechanism. Consequently, different RNA platforms are being developed for distinct classes of pathogenic variants, ranging from aberrant splicing and haploinsufficiency to gain-of-function variants and epigenetic silencing. The current landscape of representative RNA-based therapeutic strategies and their stages of clinical development is summarized in Table 1 [110,111].

### 5.1. Spinal Muscular Atrophy

SMA represents the most successful clinical application of RNA-based therapeutics in neurological disease and established the first clinical proof of concept for transcript-directed therapy in the CNS. The development of targeted RNA therapies for SMA demonstrated that correction of disease-associated RNA processing defects can fundamentally alter disease progression, improve functional outcomes, and extend survival in patients with severe genetic disorders. As such, SMA has established a paradigm for the development of RNA-based interventions across a broad range of neurodevelopmental and neurogenetic conditions [54,55].

SMA is an autosomal recessive disorder caused by biallelic loss-of-function variants in the *survival motor neuron 1* (*SMN1*) gene on chromosome 5q13. Deficiency of SMN protein results in progressive degeneration of lower motor neurons within the anterior horn of the spinal cord, leading to muscle weakness, hypotonia, respiratory compromise, and, in severe cases, early mortality. Disease severity varies considerably, ranging from severe infantile-onset forms to milder phenotypes presenting later in childhood or adulthood [126].

A distinctive feature of SMA biology is the presence of the paralogous *SMN2* gene, which differs from *SMN1* by only a few nucleotides. A critical C-to-T transition in exon 7 disrupts normal splicing and promotes exon skipping during pre-mRNA processing. As a result, the majority of *SMN2* transcripts encode a truncated and unstable protein, whereas only a small fraction generates functional SMN protein. Importantly, the number of *SMN2* copies influences residual SMN production and is a major determinant of disease severity. This unique genetic architecture provided a compelling therapeutic target for RNA-based splicing correction [127,128].

The recognition that increasing exon 7 inclusion within *SMN2* transcripts could restore functional SMN protein production led to the development of nusinersen, a splice-switching antisense oligonucleotide. Nusinersen binds to an intronic splicing silencer within *SMN2* pre-mRNA, promoting exon 7 retention and enhancing synthesis of full-length SMN protein. By directly targeting the molecular mechanism responsible for SMN deficiency, nusinersen became one of the first therapies to successfully modify disease biology through manipulation of RNA processing [129,130].

Clinical studies demonstrated substantial therapeutic benefits across multiple SMA populations. In infants with early-onset disease, nusinersen treatment significantly improved motor milestone acquisition, event-free survival, and overall survival compared with the natural history of the disorder. Similar benefits were observed in later-onset SMA, where treatment resulted in improved motor function and stabilization of disease progression. Notably, outcomes have been most striking in presymptomatically treated infants, many of whom achieve developmental milestones that would be highly unlikely in untreated disease. These observations have underscored the importance of early therapeutic intervention and have contributed to the widespread implementation of newborn screening programs for SMA [112,131].

The success of nusinersen provided the first definitive proof-of-concept that RNA-targeted therapies can produce meaningful clinical benefits in severe neurogenetic disorders. Beyond transforming SMA from a largely supportive-care condition into a treatable disease, it established a framework for the development of RNA-based therapies targeting other monogenic neurological disorders. The SMA experience demonstrated that detailed understanding of disease-specific molecular mechanisms can be translated into highly effective precision therapies capable of modifying the natural history of disease [132].

### 5.2. Rett Syndrome

RTT is a severe X-linked NDD that predominantly affects females and is characterized by developmental regression following an initial period of apparently typical development. Core clinical manifestations include loss of acquired language and purposeful hand use, intellectual disability, seizures, autistic features, gait abnormalities, autonomic dysfunction, and stereotypic hand movements. With an estimated prevalence of approximately 1 in 10,000–15,000 live female births, RTT is among the best-characterized monogenic NDDs and represents an important model for the development of precision molecular therapies [133,134].

The disorder is caused in more than 95% of classic cases by pathogenic variants in the *methyl-CpG-binding protein 2* (*MECP2*) gene on chromosome Xq28. *MECP2* encodes a multifunctional chromatin-associated protein that regulates transcription, chromatin organization, and activity-dependent gene expression in neurons. Because MECP2 controls the expression of numerous downstream genes involved in synaptic development and neuronal maturation, disruption of its function results in widespread abnormalities of neuronal connectivity and network activity [135,136,137].

A defining feature of RTT is the requirement for tightly regulated *MECP2* expression. While reduced MECP2 activity causes RTT, increased gene dosage results in MECP2 duplication syndrome, a distinct neurodevelopmental disorder with severe neurological manifestations. Consequently, therapeutic strategies must restore physiological protein levels without inducing overexpression, making dosage control a central challenge in the development of disease-modifying therapies [135,137].

This unique biology provides a strong rationale for RNA-based therapeutic approaches, which enable modulation of endogenous gene expression in a controlled and potentially reversible manner. Among these, antisense oligonucleotides (ASOs) are currently the most advanced RNA platform under investigation. Experimental studies have demonstrated that ASOs can precisely reduce excess *MECP2* expression in models of MECP2 duplication syndrome, providing proof-of-concept for dose-dependent transcript modulation. In RTT, ASO-based strategies are also being explored to regulate pathways that influence *MECP2* expression and to achieve controlled restoration of physiological protein levels. Although these approaches remain largely preclinical, they provide proof of concept for dose-dependent modulation of MECP2 expression [63,138]. More recently, modulation of *MECP2* alternative splicing has emerged as another preclinical strategy to restore physiologically relevant MeCP2 expression in RTT models [113].

RNA editing technologies offer an alternative mutation-specific strategy for RTT. Because many pathogenic *MECP2* variants are single-nucleotide substitutions, programmable ADAR-mediated editing and RNA-targeting CRISPR systems could theoretically correct mutant transcripts while preserving the endogenous pattern of gene regulation. Proof-of-concept studies have demonstrated successful editing of disease-associated variants in cellular and animal models; however, these technologies remain at an early stage of development, and significant challenges related to editing efficiency, specificity, and CNS delivery must be overcome before clinical translation becomes feasible [90,139].

Messenger RNA-based therapies have also been proposed as a means of transient protein replacement. Delivery of synthetic MECP2 transcripts could restore protein expression without genomic integration, offering a reversible alternative to DNA-based gene replacement. Nevertheless, the narrow therapeutic window associated with *MECP2* dosage makes this approach particularly challenging, as therapeutic benefit depends on achieving precise control over transcript delivery, translation, and duration of expression [81].

Preclinical studies have provided compelling evidence that neurological dysfunction associated with *MECP2* deficiency is at least partially reversible. Restoration of *MECP2* expression in symptomatic mouse models improves motor function, behavior, respiratory abnormalities, and survival, demonstrating that mature neuronal circuits retain substantial functional plasticity. These findings have fundamentally changed the therapeutic outlook for RTT by showing that meaningful neurological improvement remains possible even after symptom onset. Building on this biological rationale, several molecular therapies—including RNA- and DNA-based approaches—have entered early clinical development, although no RNA-based therapy has yet demonstrated clinical efficacy in patients [84,140].

Several important challenges continue to limit the clinical translation of RNA therapeutics for RTT. Effective treatment will require widespread delivery to neurons throughout the central nervous system, maintenance of physiological MECP2 expression over time, careful evaluation of long-term safety, and consideration of interindividual variability arising from mutation-specific effects and X-chromosome inactivation. Determining the optimal timing of intervention is also likely to be critical, given the dynamic developmental trajectory of the disorder [135,140].

### 5.3. Angelman Syndrome

AS is a rare neurogenetic disorder characterized by severe developmental delay, intellectual disability, absent or severely limited speech, ataxia, epilepsy, sleep disturbances, and a distinctive behavioral phenotype that includes frequent laughter and an unusually happy demeanor. With an estimated prevalence of approximately 1 in 12,000–20,000 individuals, AS is one of the best-characterized imprinting disorders and has emerged as a leading model for the development of RNA-based precision therapies [141,142,143].

The disorder results from the loss of functional maternal expression of the *ubiquitin protein ligase E3A* (*UBE3A*) gene located within chromosome 15q11–q13. In most tissues, both parental *UBE3A* alleles are expressed; however, in mature neurons the paternal allele is epigenetically silenced by the long non-coding antisense transcript *UBE3A-ATS* (*UBE3A* antisense transcript), leaving the maternal allele as the primary source of neuronal UBE3A protein. Consequently, maternal deletions, pathogenic variants, imprinting defects, or paternal uniparental disomy all result in a marked reduction in neuronal UBE3A expression [40,144].

This unique molecular mechanism provides an exceptional therapeutic opportunity because affected neurons retain an intact paternal *UBE3A* allele that can potentially be reactivated. Rather than replacing the missing gene, RNA-based therapies aim to suppress *UBE3A-ATS*, thereby releasing transcriptional silencing and restoring endogenous paternal *UBE3A* expression. This strategy enables physiological regulation of endogenous UBE3A expression without the need for exogenous gene replacement [40,145].

The encouraging results obtained in experimental models have led to the development of multiple clinical programs evaluating ASO-mediated paternal allele reactivation. Early-phase clinical studies have demonstrated that intrathecal administration is feasible and have provided evidence of central nervous system target engagement, supporting the biological validity of this therapeutic approach [61]. However, whether these molecular effects translate into meaningful and sustained clinical improvement remains under active investigation. Several ASO candidates have now progressed into late-stage clinical development, reflecting the maturity of this therapeutic strategy, although no RNA-based treatment has yet demonstrated definitive clinical efficacy or received regulatory approval for AS. Current studies are also evaluating whether treatment initiated during early developmental stages confers greater benefit by targeting critical periods of neuronal maturation and circuit formation [146].

Despite its strong biological rationale, several important challenges remain. Repeated intrathecal administration is currently required because of limited penetration across the BBB, and the durability of paternal *UBE3A* reactivation following treatment has not yet been fully established. Additional considerations include optimization of dosing regimens, identification of sensitive biomarkers of therapeutic response, determination of the optimal therapeutic window, and assessment of long-term safety. Furthermore, the extent to which established cognitive and behavioral deficits remain reversible after critical periods of neurodevelopment continues to be an important question for ongoing clinical investigation [147].

Beyond ASO-mediated paternal allele unsilencing, additional RNA-based strategies remain at an earlier stage of development. RNA editing technologies capable of correcting pathogenic *UBE3A* variants or modulating regulatory transcripts, as well as programmable transcriptome engineering approaches targeting imprinting mechanisms, have shown conceptual promise but remain largely experimental. These emerging platforms may broaden the range of molecular interventions available for distinct genetic subtypes of AS [97,103,148].

### 5.4. Fragile X Syndrome

FXS is the most common inherited cause of intellectual disability and one of the leading monogenic causes of autism spectrum disorder. The disorder affects approximately 1 in 4000–7000 males and 1 in 6000–11,000 females, with generally greater clinical severity in males because of its X-linked inheritance. The clinical phenotype includes intellectual disability, language impairment, anxiety, attention-deficit/hyperactivity disorder, autistic features, seizures, and characteristic behavioral abnormalities, reflecting widespread disruption of neuronal development and synaptic function [149,150].

FXS is caused by expansion of a CGG trinucleotide repeat within the 5′ untranslated region of the *fragile X messenger ribonucleoprotein 1* (*FMR1*) gene on chromosome Xq27.3. Whereas normal alleles contain fewer than 45 CGG repeats, full mutations exceeding approximately 200 repeats trigger extensive DNA methylation and chromatin remodeling, resulting in transcriptional silencing of *FMR1*. Consequently, affected individuals exhibit little or no production of FMRP, an RNA-binding protein that regulates mRNA transport, stability, and local translation at neuronal synapses [150,151].

Loss of FMRP disrupts activity-dependent protein synthesis and alters the expression of numerous genes involved in dendritic spine maturation, synaptic plasticity, and neuronal connectivity. Because FMRP regulates a broad network of neuronal transcripts rather than a single signaling pathway, its deficiency produces widespread abnormalities in synaptic function and circuit development. This complex molecular pathology distinguishes FXS from many monogenic NDDs in which correction of a single downstream defect may be sufficient to achieve therapeutic benefit [151,152,153].

The well-defined molecular basis of FXS provides a compelling rationale for RNA-based therapeutic approaches. Unlike disorders caused by structural gene disruption, however, the principal challenge in FXS is reactivation of an epigenetically silenced *FMR1* locus rather than correction of a mutant transcript. RNA-based strategies have therefore focused on restoring endogenous *FMR1* expression through modulation of regulatory RNAs or epigenetic mechanisms that maintain gene silencing. Although antisense oligonucleotides and other RNA-directed approaches have shown encouraging proof-of-concept in experimental systems, these strategies remain at an early stage of development and have not yet demonstrated clinical efficacy [42,115].

Messenger RNA replacement has also been proposed as a potential strategy to restore FMRP expression independently of endogenous gene reactivation. Delivery of synthetic *FMR1* mRNA could theoretically compensate for protein deficiency while avoiding permanent genomic modification. However, the large size of the transcript, the need for efficient delivery throughout the central nervous system, and the requirement for sustained yet physiologically regulated protein expression present substantial technical challenges. Whether transient restoration of FMRP can adequately rescue established abnormalities of neuronal circuitry also remains uncertain [80,81,87,153].

Other RNA-based approaches have primarily focused on modulating downstream molecular pathways disrupted by FMRP deficiency. Experimental studies have also identified dysregulated microRNA networks as important contributors to abnormal synaptic development, suggesting that RNA-based modulation of non-coding RNAs may represent an additional therapeutic strategy [144]. RNA interference and related technologies have been used experimentally to investigate signaling networks involving metabotropic glutamate receptor (mGluR), ERK, mTOR, and other regulators of synaptic protein synthesis. Although these studies have provided important mechanistic insights and identified potential therapeutic targets, RNA interference has largely served as a research tool rather than a clinically advanced therapeutic strategy for FXS [116,154].

Despite substantial advances in understanding disease pathogenesis, translation of RNA therapeutics into clinical application has proven considerably more challenging than in disorders such as SMA or AS. Reactivation of a heavily methylated *FMR1* locus, efficient delivery to widespread neuronal populations, and restoration of physiological FMRP expression remain major scientific and technical obstacles. In addition, many neurodevelopmental abnormalities arise during early brain development, raising important questions regarding the optimal timing of intervention and the extent to which established cognitive and behavioral deficits can be reversed [150,151]. At present, no RNA-based therapy has achieved clinical validation in FXS, and substantial preclinical and translational work remains necessary before these approaches can be translated into effective disease-modifying treatments [115,117,155].

### 5.5. Emerging RNA Therapeutics for Monogenic Neurodevelopmental Disorders

Beyond disorders in which RNA therapeutics have already demonstrated clinical benefit or advanced into late-stage clinical development, a growing number of monogenic NDDs are emerging as candidates for transcript-directed precision medicine. Advances in next-generation sequencing, disease modeling, and RNA engineering have enabled the identification of pathogenic mechanisms that may be amenable to ASOs, RNA interference, mRNA replacement, or RNA editing. Although most of these programs remain at the preclinical stage, they illustrate the expanding scope of RNA therapeutics for genetically defined neurological disease [110,111].

#### 5.5.1. Developmental and Epileptic Encephalopathies

Among emerging indications, developmental and epileptic encephalopathies (DEEs) caused by pathogenic variants in neuronal ion channel genes represent some of the most advanced targets for RNA-based therapeutic development. Because many of these disorders result from well-defined gain-of-function or loss-of-function mechanisms, RNA therapeutics can be designed to selectively suppress or restore gene expression according to the underlying molecular defect [65,156].

The *SCN2A* gene, which encodes the voltage-gated sodium channel Nav1.2, exemplifies this genotype-directed therapeutic paradigm. Gain-of-function variants are typically associated with neonatal or infantile epileptic encephalopathy and constitute attractive candidates for allele-selective transcript suppression using antisense oligonucleotides or RNA interference [64,90]. Conversely, loss-of-function variants, which more commonly present with autism spectrum disorder, intellectual disability, and later-onset epilepsy, may ultimately benefit from transcript-enhancing or mRNA replacement strategies [17]. Robust preclinical studies have demonstrated the feasibility of ASO-mediated modulation of *SCN2A* expression, and highly individualized RNA-based therapeutic approaches have recently entered early translational evaluation under carefully controlled clinical circumstances. Although these initial experiences support the feasibility of personalized RNA medicine, clinical efficacy has not yet been established, and larger studies will be required to determine long-term safety and therapeutic benefit [64,118].

Pathogenic gain-of-function variants in *KCNT1* represent another promising target for RNA-directed therapy. *KCNT1* encodes the sodium-activated potassium channel KNa1.1, and excessive channel activity underlies severe developmental and epileptic encephalopathies, including epilepsy of infancy with migrating focal seizures and autosomal dominant sleep-related hypermotor epilepsy [42]. Preclinical studies have shown that ASO-mediated reduction in *KCNT1* expression decreases seizure frequency, improves survival, and ameliorates neurological phenotypes in animal models. More recently, limited early human experience has demonstrated the feasibility of individualized ASO treatment in a small number of patients with severe drug-resistant disease. While these observations provide encouraging translational signals, they remain exploratory and should not be interpreted as evidence of established clinical efficacy [66,119,157].

The closely related sodium channel disorder associated with *SCN8A* has likewise emerged as a strong preclinical candidate for RNA therapeutics. Experimental ASOs have demonstrated significant reductions in seizure burden and improved survival in multiple animal models, including treatment initiated after seizure onset. These studies provide compelling proof-of-concept that transcript suppression may modify disease progression in *SCN8A*-related epileptic encephalopathy. However, clinical evaluation has not yet begun, and translation will require further optimization of allele specificity, dosing, and long-term safety [120,158].

#### 5.5.2. Synaptopathies

Among disorders caused by impaired synaptic development and function, *SYNGAP1* haploinsufficiency represents one of the strongest biological candidates for RNA-based therapeutic intervention. *SYNGAP1*-related intellectual disability is characterized by developmental delay, epilepsy, autism spectrum disorder, and cognitive impairment resulting from reduced expression of the SynGAP protein [16]. Because disease pathogenesis is primarily driven by haploinsufficiency, relatively modest increases in endogenous protein expression may be sufficient to improve neuronal function [85]. Experimental strategies including splice-modulating antisense oligonucleotides and transcript-enhancing approaches have demonstrated encouraging results in cellular and animal models, supporting continued preclinical development. Nevertheless, no RNA-based therapy has yet entered clinical evaluation, and important challenges remain regarding efficient CNS delivery, therapeutic timing, and sustained restoration of physiological SynGAP expression [121,159].

Pathogenic variants in *SHANK3* cause Phelan–McDermid syndrome, another synaptopathy characterized by intellectual disability, autism spectrum disorder, hypotonia, and language impairment. Although restoration of SHANK3 expression represents an attractive therapeutic objective, RNA-based approaches remain at an early stage of investigation [160]. Furthermore, the large size of the *SHANK3* transcript, the existence of multiple alternatively spliced isoforms, and the complex developmental regulation of SHANK3 expression present substantial technical challenges for RNA therapeutics. Notably, the most advanced disease-modifying therapeutic programs targeting *SHANK3* currently involve gene replacement rather than RNA-based interventions, and RNA-directed strategies should therefore be regarded as experimental [122,161].

#### 5.5.3. Other Emerging Therapeutic Targets

Additional monogenic NDDs involving genes such as *STXBP1*, *MEF2C*, *GRIN2B*, *CACNA1C*, and *CHD8* are increasingly being explored as potential candidates for RNA-based therapeutics. These genes participate in diverse biological processes, including neurotransmitter release, transcriptional regulation, synaptic signaling, calcium homeostasis, and chromatin remodeling [23,42]. Recent experimental studies further support the therapeutic potential of RNA-directed approaches targeting STXBP1, CACNA1C, and CHD8 [123,124,125].

Progress in patient-derived induced pluripotent stem cell models, brain organoids, and genetically engineered animal models has substantially improved understanding of disease mechanisms and accelerated evaluation of candidate RNA therapeutics. Nevertheless, therapeutic development for these disorders remains at an early stage, with most RNA-based approaches yet to progress beyond preclinical investigation [147,162].

## 6. Delivery Strategies for Central Nervous System-Targeted RNA Therapeutics

The rapid expansion of RNA-based therapeutics has demonstrated that successful treatment of genetic NDDs depends on more than the identification of an appropriate molecular target. Clinical translation requires the convergence of three interdependent elements: a well-defined disease mechanism, an RNA modality capable of correcting the underlying molecular defect, and a delivery system that can achieve safe and efficient distribution within the central nervous system (CNS). The experience gained from disorders such as SMA and, more recently, AS illustrates that advances in RNA engineering alone are insufficient if therapeutic molecules cannot reach the appropriate neuronal populations at adequate concentrations. Consequently, delivery has emerged as one of the principal determinants of the translational maturity of RNA therapeutics for NDDs [80,110].

The CNS presents unique challenges for nucleic acid delivery. The BBB, together with the specialized architecture of the neurovascular unit, restricts the passage of large, hydrophilic, and negatively charged RNA molecules from the systemic circulation into the brain parenchyma. In addition, unmodified RNAs are susceptible to rapid nuclease degradation, exhibit poor cellular uptake, and may activate innate immune pathways. Effective CNS-directed RNA therapeutics therefore require not only chemically optimized RNA molecules but also delivery strategies capable of overcoming extracellular barriers, promoting cellular internalization, and ensuring intracellular bioavailability [76,88].

Importantly, delivery requirements differ substantially among RNA therapeutic platforms. Antisense oligonucleotides (ASOs) have achieved the greatest degree of clinical translation because they can function following direct administration into the cerebrospinal fluid and do not require sustained intracellular protein expression [163]. In contrast, siRNAs require efficient cytoplasmic delivery and endosomal escape to engage the RNA-induced silencing complex, whereas mRNA therapeutics depend on successful intracellular translation of relatively large RNA molecules [76,81,89]. RNA editing technologies present even greater delivery demands because therapeutic efficacy often requires coordinated delivery of guide RNAs together with programmable editing machinery. These modality-specific requirements have become an important determinant of the relative clinical maturity of RNA therapeutic platforms [91].

### 6.1. Direct Cerebrospinal Fluid Administration: The Current Clinical Benchmark

Direct administration into the cerebrospinal fluid currently represents the most clinically established strategy for CNS delivery of RNA therapeutics. Intrathecal administration bypasses the BBB and enables widespread distribution within the cerebrospinal fluid, although uniform penetration into all brain regions and therapeutically relevant neuronal populations remains challenging [76]. The approval of nusinersen for SMA established this approach as the first clinically validated delivery platform for RNA therapeutics in neurological disease and demonstrated that repeated intrathecal administration can achieve durable therapeutic benefit. The same delivery strategy has subsequently been adopted by multiple ASO programs targeting NDDs, including AS and other genetically defined neurological diseases [54,61,112].

Intracerebroventricular administration provides an alternative route capable of achieving broader cerebrospinal fluid distribution in selected experimental settings and certain pediatric indications. However, both approaches remain invasive and require specialized clinical expertise. Repeated lumbar punctures or ventricular access procedures increase treatment burden and may be associated with complications including cerebrospinal fluid leakage, infection, bleeding, and post-procedural headache. Nevertheless, direct cerebrospinal fluid administration currently remains the reference standard against which emerging CNS delivery technologies are measured [7,146].

### 6.2. Lipid Nanoparticles and Other Non-Viral Delivery Systems

The growing interest in siRNA-, mRNA-, and RNA-editing therapeutics has intensified efforts to develop delivery systems capable of overcoming the limitations of direct cerebrospinal fluid administration. Among these, lipid nanoparticles (LNPs) have become the leading non-viral platform for RNA delivery because they protect nucleic acids from degradation, facilitate cellular uptake, and promote endosomal escape. Their clinical success in extra-CNS applications has stimulated extensive research into formulations optimized for neurological disease [7,81,87].

However, currently available LNPs remain largely optimized for hepatic delivery following systemic administration and exhibit limited penetration across the BBB. Consequently, no brain-targeted LNP platform has yet achieved established clinical use for RNA therapeutics in neurodevelopmental disorders. Accordingly, considerable efforts are focused on engineering brain-targeted nanoparticles through ligand-mediated targeting, optimization of particle composition, and enhancement of receptor-mediated transcytosis. Such strategies are particularly relevant for siRNA, mRNA, and RNA editing platforms, whose therapeutic activity depends on efficient intracellular delivery [7,50,88].

Additional non-viral systems, including biodegradable polymeric nanoparticles, dendrimers, and lipid–polymer hybrid nanocarriers, are also being investigated to improve CNS biodistribution and reduce toxicity. Although these technologies have demonstrated encouraging results in experimental models, they remain largely in the preclinical or early translational stages of development, and their safety, scalability, and reproducible CNS targeting must be established before widespread clinical application becomes feasible [87,89].

### 6.3. Delivery of RNA-Based Therapeutic Components

Certain RNA-based therapeutic strategies require prolonged intracellular expression of guide RNAs or RNA editing components that cannot be achieved through repeated administration of synthetic oligonucleotides alone. In these settings, viral vectors, most commonly adeno-associated viruses (AAVs), may serve as delivery vehicles for RNA-related therapeutic components rather than as gene replacement therapies. Their neuronal tropism and ability to support sustained expression make them attractive for selected RNA editing and transcriptome engineering applications [7,90,91].

Nevertheless, the use of viral delivery introduces additional considerations, including limited packaging capacity, pre-existing immunity, manufacturing complexity, and prolonged persistence of therapeutic components that may diminish one of the principal advantages of RNA medicine—its reversibility. Consequently, viral systems currently occupy a more specialized role within the broader landscape of RNA therapeutics and are primarily being explored in experimental applications requiring long-term intracellular expression [90,91].

Extracellular vesicles, particularly exosomes, represent another emerging delivery platform. Their intrinsic biocompatibility, low immunogenicity, and capacity to transport endogenous RNA molecules have generated considerable interest as vehicles for ASOs, siRNAs, mRNAs, and RNA editing components. Although engineered extracellular vesicles have shown encouraging preclinical results, important challenges related to cargo loading, manufacturing scalability, targeting specificity, and reproducibility remain unresolved, and clinical translation is still at an early stage [89].

### 6.4. Delivery as a Determinant of Translational Success

The experience accumulated across RNA therapeutics demonstrates that successful clinical translation depends on the coordinated optimization of three interdependent factors: selection of an appropriate molecular target, identification of the most suitable RNA therapeutic modality, and development of a delivery strategy capable of achieving safe, efficient, and durable CNS exposure. Deficiencies in any one of these components may limit therapeutic efficacy irrespective of advances in the others [90,91,92].

Current clinical experience reflects these principles. ASO-based therapies have achieved the greatest degree of clinical translation, whereas broader implementation of siRNA, mRNA, and RNA editing technologies will depend on continued advances in CNS-targeted delivery capable of supporting efficient, cell-specific intracellular activity [80,90,91].

## 7. Key Challenges and Future Directions in RNA-Based Therapeutics

Despite remarkable advances in RNA medicine over the past decade, several biological, technological, and clinical challenges continue to limit the broader implementation of RNA-based therapeutics for genetic NDDs. While clinical proof-of-concept has been established in selected conditions, most notably SMA, translation to the wider spectrum of monogenic NDDs remains considerably more complex. The principal obstacle is no longer the availability of therapeutic RNA modalities themselves, but the need to integrate disease biology, RNA engineering, delivery technologies, and clinical implementation into a coherent therapeutic strategy. Addressing these interconnected bottlenecks will determine the future success of RNA-based precision medicine for neurological disease [42,80,110].

### 7.1. Biological and Patient-Level Constraints

The effectiveness of RNA therapeutics is fundamentally influenced by disease biology and patient-specific factors. One of the most important determinants is the timing of therapeutic intervention. Brain development follows tightly regulated temporal windows during which neuronal proliferation, migration, synaptogenesis, and circuit refinement occur. Many monogenic NDDs begin to disrupt these processes prenatally or during early infancy, often before clinical diagnosis is established. Consequently, treatment initiated after substantial circuit abnormalities have developed may provide only partial functional recovery. Although disorders such as SMA and RTT have demonstrated that aspects of neurological dysfunction remain reversible, the extent to which established neurodevelopmental abnormalities can be corrected remains uncertain [23,115,147].

Clinical and molecular heterogeneity further complicate therapeutic development. Individuals carrying pathogenic variants in the same gene frequently exhibit marked differences in disease severity, age at onset, neurological manifestations, and therapeutic responsiveness. Such variability emphasizes the importance of genotype-informed patient stratification and individualized therapeutic approaches rather than uniform treatment strategies [111,117,156].

Another major limitation is the lack of robust biomarkers capable of monitoring target engagement and predicting therapeutic response. Current outcome measures often rely on developmental assessments or functional rating scales that require prolonged follow-up and may inadequately capture early biological effects. The development of molecular, imaging, electrophysiological, and other pharmacodynamic biomarkers will therefore be essential for accelerating clinical evaluation and optimizing treatment decisions [147,158].

For many disorders, therapeutic specificity also remains a critical consideration. Allele-specific suppression is particularly important for dominant gain-of-function disorders, whereas dosage-sensitive conditions require restoration of physiological gene expression without inducing either under- or overexpression. Achieving this level of molecular precision remains a major challenge for RNA therapeutic design [90,91,92].

### 7.2. Translational Barriers from Experimental Models to Clinical Practice

Even when compelling biological rationale exists, successful translation from experimental systems to human disease remains challenging. Animal models have been indispensable for defining disease mechanisms and establishing proof-of-concept for RNA therapeutics, yet they often fail to reproduce the complexity of human neurodevelopment, genetic diversity, and disease progression. Species-specific differences in RNA processing, alternative splicing, epigenetic regulation, and neuronal maturation may substantially influence therapeutic responses and contribute to discrepancies between preclinical and clinical findings [80,111].

Human-induced pluripotent stem cell-derived neurons, brain organoids, and advanced three-dimensional culture systems have begun to address some of these limitations by providing disease-relevant human models for therapeutic evaluation. Nevertheless, these systems remain simplified representations of the developing brain and cannot fully recapitulate long-range neuronal connectivity, vascular interactions, or the complexity of the in vivo microenvironment [110,111].

Clinical implementation presents additional challenges. Most monogenic NDDs are rare, limiting patient recruitment and reducing statistical power in clinical trials. Furthermore, disease progression is frequently heterogeneous, and conventional clinical endpoints may inadequately capture meaningful improvements in cognition, communication, adaptive behavior, or quality of life. Comprehensive natural-history studies, standardized outcome measures, and validated biomarkers will therefore be essential for accurately evaluating therapeutic efficacy and facilitating regulatory approval [80,136,153].

### 7.3. Technological Innovations Addressing Current Limitations

Continued advances in RNA engineering are expected to alleviate many of the limitations associated with first-generation therapeutics. Improvements in antisense oligonucleotide chemistry have enhanced molecular stability, target affinity, tissue retention, and resistance to nuclease degradation while reducing immunogenicity and off-target interactions. These developments may improve long-term safety and reduce the frequency of therapeutic administration [80,110].

At the same time, substantial progress is being made in technologies that directly address current translational bottlenecks. Increasingly sophisticated allele-specific approaches aim to maximize therapeutic precision in dominant disorders, while advances in CNS-targeted delivery systems seek to improve neuronal uptake, tissue distribution, and intracellular bioavailability. Parallel developments in programmable RNA editing platforms continue to expand the range of pathogenic variants that may become amenable to transcript-level correction while preserving the reversible nature of RNA-therapeutics [76,91,92].

Although many of these technologies remain at the preclinical or early translational stage, their clinical value will depend on demonstrating meaningful improvements in delivery, safety, and therapeutic efficacy [80,110].

### 7.4. An Integrated Framework for RNA-Based Precision Medicine

The experience accumulated across RNA therapeutics indicates that successful clinical translation depends on substantially more than demonstration of preclinical efficacy.

Although numerous RNA-based strategies have shown encouraging results in experimental models, relatively few have progressed to clinical implementation. This discrepancy reflects the fact that individual neurodevelopmental disorders differ not only in their molecular mechanisms but also in their biological constraints, including therapeutic timing, dosage sensitivity, cellular targets, and requirements for widespread CNS delivery. Consequently, the same RNA platform may achieve markedly different translational outcomes depending on the disease context [80,110,111].

**Figure 2:**
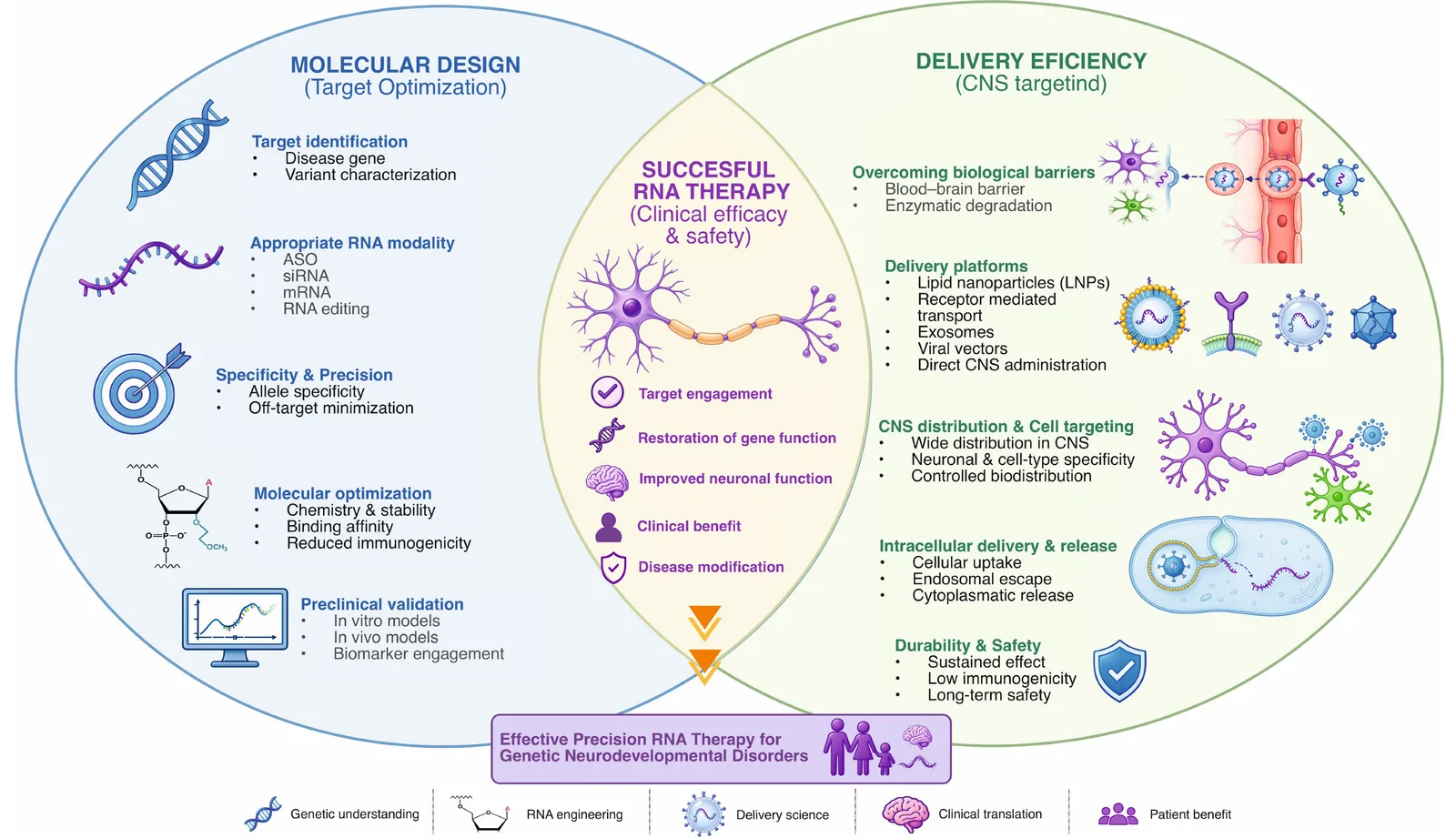
Dual bottleneck model for the clinical translation of RNA therapeutics in genetic neurodevelopmental disorders. Molecular design encompasses disease-gene selection, therapeutic RNA platform choice, sequence optimization, target specificity, and preclinical validation, whereas delivery efficiency involves overcoming biological barriers, achieving efficient central nervous system distribution, cell-specific uptake, intracellular bioavailability, and sustained therapeutic exposure. Successful clinical translation emerges from the coordinated optimization of both domains, emphasizing that advances in RNA engineering and delivery science are complementary requirements for effective precision RNA therapeutics.

Current evidence also suggests that the principal limitation is no longer the availability of RNA therapeutic platforms themselves. Antisense oligonucleotides, siRNAs, mRNA therapeutics, and RNA editing technologies collectively provide a versatile repertoire of transcript-directed interventions capable of addressing diverse pathogenic mechanisms. Instead, successful clinical development increasingly depends on selecting the appropriate platform for the underlying disease mechanism and integrating this choice with effective delivery strategies, biomarkers of target engagement, and carefully designed clinical studies [110,111]. Taken together, these observations suggest that the next phase of RNA therapeutics will be driven less by the discovery of entirely new molecular platforms than by continued refinement of existing technologies and their adaptation to disease-specific biological contexts. Advances in delivery systems, allele-specific targeting, programmable RNA engineering, biomarker development, and patient stratification are therefore expected to play a central role in expanding the range of neurodevelopmental disorders amenable to RNA-based therapies [80,110]. This integrated translational pathway is summarized in Figure 2 [80,110].

## 8. Conclusions

RNA-based therapeutics are redefining the therapeutic landscape of genetic NDDs by enabling precise modulation of gene expression at the transcript level. The clinical success of nusinersen in SMA demonstrated that RNA-directed intervention can modify the natural history of severe neurogenetic disease and has provided a foundation for the development of similar strategies across a broader spectrum of disorders. Since then, advances in antisense oligonucleotides, small interfering RNAs, messenger RNA therapeutics, RNA editing technologies, and emerging RNA-based platforms have expanded the possibilities for targeting diverse pathogenic mechanisms, including aberrant splicing, haploinsufficiency, gain-of-function variants, toxic transcripts, and dysregulated gene expression.

The translation of these approaches into effective therapies for NDDs requires more than the availability of an RNA platform. Successful intervention depends on the appropriate alignment of disease biology, therapeutic design, and efficient CNS delivery. Genotype-driven diagnosis is therefore becoming increasingly important not only for establishing molecular etiology, but also for guiding therapeutic eligibility, mechanism-based target selection, and the optimal RNA-based strategy.

Despite rapid progress, major challenges remain. The BBB, restricted CNS biodistribution, cell-type specificity, developmental timing, long-term safety, immune activation, and durability of therapeutic effects continue to limit clinical translation. These issues are particularly important in pediatric NDDs, where intervention may need to occur during critical windows of brain development and where long-term consequences of treatment must be carefully evaluated. Continued advances in RNA engineering, delivery technologies, biomarker development, and clinical trial design will be essential for transforming promising experimental approaches into safe and effective therapies.

As molecular diagnosis becomes increasingly integrated into clinical care, RNA therapeutics offer a powerful framework for precision medicine in neurodevelopmental disease. Their adaptability allows therapeutic strategies to be matched to the underlying genetic mechanism, creating opportunities for individualized and mechanism-based treatment. Although many approaches remain at an early stage, the convergence of genomic medicine, RNA engineering, and CNS-directed delivery is expected to accelerate the development of disease-modifying therapies for genetic NDDs. In this context, RNA medicine represents not only a new therapeutic class, but also a conceptual shift toward treating NDDs at their molecular origin.

## Figures and Tables

**Table 1:** Current landscape of RNA-based therapeutic strategies and stages of clinical development in representative neurodevelopmental and neurogenetic disorders.

Disorder	Gene	Disease Mechanism	RNA Therapeutic Approach	Representative Strategy	Stage of Development	References
Spinal muscular atrophy	*SMN1*/*SMN2*	Aberrant *SMN2* exon 7 splicing	ASO	Nusinersen (splice switching)	Approved	[54,55,112]
Rett syndrome	*MECP2*	Haploinsufficiency; dosage-sensitive gene	ASO, mRNA, RNA editing	*MECP2* modulation/editing	Preclinical–Early clinical	[113]
Angelman syndrome	*UBE3A*	Genomic imprinting (paternal allele silencing)	ASO	UBE3A-ATS unsilencing	Phase II–III clinical trials	[61,114]
Fragile X syndrome	*FMR1*	Epigenetic silencing	RNA directed/epigenetic reactivation	*FMR1* reactivation	Experimental	[115,116,117]
SCN2A-DEE	*SCN2A*	Gain-of-function/haploinsufficiency	ASO	Allele-specific suppression or transcript suppression	Individualized translational studies	[64,118]
KCNT1-DEE	*KCNT1*	Gain-of-function	ASO	Transcript suppression	Early clinical experience	[66,119]
SCN8A-DEE	*SCN8A*	Gain-of-function	ASO	Transcript suppression	Preclinical	[65,120]
SYNGAP1-ID	*SYNGAP1*	Haploinsufficiency	ASO	Splice modulation/transcript enhancement	Preclinical	[121]
Phelan–McDermid syndrome	*SHANK3*	Haploinsufficiency	ASO (experimental), gene regulation	Endogenous *SHANK3* upregulation	In vitro/Preclinical	[122]
Other monogenic NDDs	*STXBP1*, *CACNA1C*, *CHD8*	Diverse mechanisms/Haploinsufficiency/gain-of-function	ASO, RNA-based translational enhancement	Splice modulation/transcript or protein enhancement	Exploratory/Preclinical	[123,124,125]

ASO Antisense oligonucleotide; mRNA Messenger RNA; siRNA Small interfering RNA; NDDs Neuro-developmental disorders.

## Data Availability

No new data were created or analyzed in this study. Data sharing is not applicable to this article.

## Abbreviations

The following abbreviations are used in this manuscript:

AAV	Adeno-associated virus
ADAR	Adenosine deaminase acting on RNA
AS	Angelman syndrome
ASD	Autism spectrum disorder
ASDs	Autism spectrum disorders
ASO	Antisense oligonucleotide
BBB	Blood–brain barrier
circRNA	circular RNAs
CGG	Cytosine–guanine–guanine trinucleotide repeat
CNS	Central nervous system
COVID-19	Coronavirus disease 2019
CRISPR	Clustered Regularly Interspaced Short Palindromic Repeats
DEE	Developmental and epileptic encephalopathy
DEEs	Developmental and epileptic encephalopathies
DNA	Deoxyribonucleic acid
ERK	Extracellular signal-regulated kinase
FMRP	Fragile X messenger ribonucleoprotein
FXS	Fragile X syndrome
ID	Intellectual disability
lncRNA	Long non-coding RNA
LNA	Locked nucleic acid
LNP	Lipid nanoparticle
mGluR	Metabotropic glutamate receptor
miRNA	MicroRNA
mRNA	Messenger RNA
mTOR	Mechanistic target of rapamycin
NDD	Neurodevelopmental disorder
NDDs	Neurodevelopmental disorders
NGS	Next-generation sequencing
NMD	Nonsense-mediated mRNA decay
RNA	Ribonucleic acid
RNAa	RNA activation
RNAi	RNA interference
RISC	RNA-induced silencing complex
RTT	Rett syndrome
saRNA	Small activating RNA
SCN	Sodium voltage-gated channel (gene family prefix, e.g., *SCN2A*, *SCN8A*)
siRNA	Small interfering RNA
SMA	Spinal muscular atrophy
SMN	Survival motor neuron
UBE3A-ATS	UBE3A antisense transcript
UTR	Untranslated region
WES	Whole-exome sequencing
WGS	Whole-genome sequencing
